# Supplementary material for: Are carbon emissions trading and green financial instruments synergistic? -Comprehensive quantitative research based on content analysis
Source: PLoS One. 2024 Mar 7;19(3):e0298601. doi: 10.1371/journal.pone.0298601 (PMC10919601; doi:10.1371/journal.pone.0298601)
Supplement: S1 Data — (ZIP) [file pone.0298601.s001.zip › Data/地级市Z_F工作报告.zip!/╣π╢1⁄2/╓╨╔╜╩╨/└┤╫╘╣1⁄2╓┌║┼í╛┬φ┐╦╩2╛▌═°í┐_2009.docx]

2008年工作回顾

　　2008年是我市积极应对严峻考验，推进科学发展取得新成绩的一年。在省委、省政府和市委的正确领导下，在市人大及其常委会和市政协的监督支持下，市政府高举中国特色社会主义伟大旗帜，以邓小平理论和“三个代表”重要思想为指导，以科学发展观统领发展全局，认真贯彻党的十七大及十七届三中全会精神，坚持解放思想，主动迎难而上，切实转变发展方式，保持经济平稳较快发展，社会事业不断进步，城市综合实力增强，人民生活持续改善。

　　经济实现平稳较快发展。全市预计生产总值1409亿元，增长11.1%，户籍人口人均生产总值96506元（常住人口人均生产总值56034元）。全社会工业总产值4091亿元，增长14.1%（现价）。财税金融运行平稳，地方一般预算收入突破100亿元，增长16.3%。国地两税收入234.3亿元，增长17.6%。人民币各项存款余额1769.8亿元，比年初增长17.2%。投资消费协调性增强。全社会固定资产投资445.1亿元，增长11.5%。社会消费品零售总额476.9亿元，增长20.5%。

　　现代产业体系建设初显成效。产业结构不断优化，三次产业比例调整为3.2:60.2:36.6。投入扶持产业发展专项资金2.4亿元。实现工业增加值预计810亿元，增长12.8%（现价）。工业结构高级化和适度重型化进程加快，重工业比重提高1.3个百分点，装备制造业产值占工业总产值24.5%。大企业大项目建设提速，中船制造项目落户中山，船舶工业制造基地、华南现代中医药城等加快推进，明阳风电设备、天乙铜材等项目投产。高新技术产品产值突破1200亿元，占工业总产值30.5%。传统特色产业巩固发展，产业集群升级创新加快，全市建有国家级产业基地26个。服务业增效明显，实现增加值预计515亿元，增长14%。传统服务业保持平稳增长，现代服务业比重明显提高。一批有实力的金融保险机构落户中山，全省首家村镇银行在小榄开业。新增上市及整体上市企业2家。中心商务区和商圈建设加快。会展业成效显著，市级特色专业会展发展到11个。首次举办游戏游艺博览会和绿博会，带动新产业形成。城市接待过夜游客531万人次，旅游业总收入97.3亿元。农业农村经济稳步增长，实现农业总产值76.3亿元，增长3.6%。

　　自主创新能力不断增强。全市有9项科研成果获省科技奖。拥有省级以上各类工程技术研发中心16家，建成国家灯具质量监测检验中心，小榄生产力促进中心成为国家级示范生产力促进中心。品牌战略深入推进，新增省级以上名牌名标64个。围绕支柱产业、关键技术和共性技术组建产学研联盟，实施合作项目200余项。拥有16项产业集群联盟标准。加强地理标志产品保护，推进服务业标准化工作。获评全国知识产权试点先进市，第3次成为全国科技进步考核先进市。

　　内外源经济协调发展。民营经济发展加快，规模以上民营工业总产值1338亿元，增长21.8%，高于规模以上工业总产值增速10.1个百分点，民营经济对全市经济的贡献明显增强。进出口贸易总值预计266亿美元，增长8%，其中出口总值预计190.5亿美元，增长10.1%。招商引资取得新成绩，实际利用外资8.3亿美元，其中投资超千万美元项目10宗。国家批准中山建设保税物流中心，我市设立首个海关特殊监管区。首次推出股权招商，合同引进资金13.2亿元。有序推进产业和劳动力“双转移”，中山河源、中山肇庆大旺产业转移园区成为全省首批产业转移示范性园区。加大省内城市劳务合作，促进劳动力转移就业。

　　统筹城乡发展步伐加快。坚持实施强农惠农政策，市财政投入4.3亿元促进城乡协调发展。在全省率先实施政策性农业保险。城乡公共服务均等化加快推进，建成农村候车亭220个，拥有农村（社区）卫生服务站250间，建设硬底化主干农路250公里，67%欠发达村实现集体经济年纯收入超100万元。现代农业迅速发展，建有6个现代农业示范园区。启动全市村庄规划，加快城中村改造，建成农民居住小区18个，农民居住环境不断改善。城乡差距进一步缩小。

　　基础设施建设更加完善。重点项目建设完成投资81.3亿元，圆满完成全年计划。古神公路、市博览中心、博爱医院儿科大楼、第二人民医院、新泰安市场、珍家山污水处理收集系统一期、220千伏菊城和宝山输变电、嘉明电厂二期首台机组及输出线路等重点工程投入使用，新岐江公路、新沙水公路、福源路、中山港二期、广珠轻轨、新中医院、人民医院杨郭恩慈大楼、北部和南部组团垃圾综合处理基地、500千伏桂山输变电等项目加快推进。投放和更新公交车325辆，新增公路里程55.6公里。干堤达标加固30公里。创建全国生态市通过省级验收和国家技术核查。污水处理厂建设如期推进，建成全省第一批污染源在线监控系统，单位生产总值能耗下降3.9%，二氧化硫和化学需氧量排放量分别下降8.4%和19%。完成山地林相改造1.3万亩，森林覆盖率达16.3%。城市综合管理水平不断提高。

　　重点领域改革成效显著。国有资产改革向纵深发展，市属国有企业实现净利润5.5亿元，净资产收益率9.3%。深化农业农村改革，推进土地承包经营权流转，41.4%村（居）完成村级统一核算改革。行政审批制度改革不断深化，41个部门517个审批事项实现网上流转办理。建立重点项目审批“绿色通道”，努力缩短审批时间。公共财政管理体制改革成效明显，对三角、横栏、黄圃镇实行一级财政管理，对阜沙、民众、大涌、神湾镇加大转移支付力度。规范镇区财务管理，土地出让收支纳入财政预算。推进投融资体制改革，以中心粮库二期等为试点推行代建制建设。在城区开展居民用水阶梯水价试点工作。

　　社会事业全面进步。市财政投入49亿元用于公共产品和公共服务支出，占财政一般预算支出57%。实施积极就业创业政策，新增就业岗位7.1万个，启动青年创业工程，城镇登记失业率为2.1%，培训失业无业人员、自主创业人员和农民工9.5万人次。城乡一体化社会保障体系基本建立，全市参加城镇社会保险460万人次，农村基本养老保险覆盖率超过80%。全民安康工程加快推进，城乡居民住院基本医疗保险实现全覆盖，参加城乡居民门诊基本医疗保险23.5万人。市财政投入教育事业15亿元。改善中小学、幼儿园教师工资福利待遇。不断提升基础教育水平，规范学前教育管理，中小学优质学位覆盖率超过85%，高中教育水平进一步提高。普通高考录取率连续三年全省第一，高等教育毛入学率50.1%，进入普及化阶段。超常规发展职业技术教育，民办教育健康发展。市一中初中部迁建、中山纪念中学扩建、中山中专和广东理工职业学院中山校区首期工程投入使用，中山中专二期、中山职业技术学院三期、第一职业技术学校、高级技工学校黄圃校区等工程全面推进。加快文化名城建设，启动创建国家历史文化名城申报工作，孙中山故居纪念馆成为全国首批国家一级博物馆，翠亨村成为国家历史文化名村，新增2项国家非物质文化遗产。成功举办2008广东国际旅游文化节闭幕式。开展数字电视转换试点工作。大力发展慈善事业，筹建慈善总会，举行第21届慈善万人行活动，建成23间慈善捐助超市。优化人口结构，提高人口素质，人口自然增长率为4.65‰。全面落实优抚安置，连续第5次被命名为省双拥模范城。以北京奥运会为契机，推进竞技体育和全民健身运动，获得全国冠军11个，亚洲冠军1个。成功抵御严重雨雪冰冻和超强台风等自然灾害。市财政安排9000万元，社会捐款捐物2.4亿元，全力支援抗震救灾，对口援建汶川县漩口镇。

　　人民生活质量稳步提高。居民收入持续增长，城镇居民年人均可支配收入21536元，增长6%；农民人均年纯收入11001元，增长10%。采取有效措施稳定物价，发放食品、生猪屠宰、燃气、汽油等价格补贴5600万元，居民消费价格总指数升幅为5.6%。住房保障体系逐步建立，首批廉租房187套如期交付。完成第三期农村低保家庭危房改造工程。努力稳定房地产市场，市场化供应商品住宅320.4万平方米。平安中山建设顺利推进，应急体系进一步完善，建成公安视频监控点3272个，社会治安状况稳定，取得奥运安保攻坚战全面胜利。安全生产形势良好，各类安全事故宗数下降16.1%。评选“百佳流动务工人员”，流动人口服务管理不断完善。开展重点商品市场专项整治，产品质量和食品安全监管体系进一步健全。

　　政府自身建设不断加强。坚持依法治市，主动接受人大法律监督、工作监督和政协民主监督，办结关于加强农村（社区）卫生站建设的议案，办理人大代表建议102件、政协提案201件，答复率100%。深入推行政务公开，升级政府门户网站，整合建成12345行政服务热线，完善网上政务投诉统一平台。坚持市长接待日等接访制度，受理群众来信来访事项1821宗。完成村（居）委会换届选举工作，基层政权建设水平进一步提高。建设服务型政府，及时出台20多项政策促进经济发展，组织经济工作服务队深入基层，帮助企业渡难关。加强三级电子监察系统建设，行政效能提升成效明显，廉政建设和反腐败工作不断加强。深入开展“五五”普法活动，在全省率先推开规范行政处罚自由裁量权工作。制定落实科学发展观评价指标体系及考核实施办法，公务员队伍整体素质不断提高。

　　外事、侨务、港澳事务、台务、民族、宗教、审计、统计、老干、气象、国防建设、档案、地方志、妇女儿童、残疾人、人防、扶贫、老区建设等各项工作取得新成绩。

　　过去一年国内外多种不利因素叠加，面临前所未有的困难和挑战。我们坚定信心、多管齐下、有效应对，做到出手快、措施准、工作实，提高了驾驭复杂局面、推进科学发展的能力，用实际行动向我国改革开放30周年和中山升格地级市20周年献礼。我们深刻体会到：坚持解放思想，在复杂多变的国内外经济形势下出台系列政策，拉动投资，提振信心，共渡难关，是发挥政府“有形之手”作用的具体体现；坚持科学发展，不断巩固优化以集群为主要特征的产业组织形式，抵抗风险，帮扶企业，促进繁荣，是驱动发展的“经济引擎”；坚持以人为本，全面统筹城乡发展，致力民生，缩小差距，维护稳定，是经济社会平稳较快发展的“稳压器”；坚持从实际出发，把中山人民的根本利益作为所有工作的立足点，实事求是，恪尽职守，开拓进取，是我们执政为民过程中始终牢记的重要宗旨。

形势与目标

　　2009年是新中国成立60周年，也是我市推动经济社会全面转入科学发展轨道、实现“十一五”规划目标的关键之年。做好今年工作，对于抵御国际金融危机冲击、保持经济社会又好又快发展具有特殊重要的意义。

　　纵观当前国内外形势，挑战与机遇共在，困难与利好并存。从国际看，金融危机的影响继续扩散和蔓延，世界经济经历低迷和调整期，经济不稳定不确定因素增多，全球经济增长明显放缓；从国内看，我国经济经受着外部需求减少、内部周期性结构性调整的双重考验，经济社会发展面临的困难可能比我们想象的要大，经济下行风险增加；从全市看，经济外向依存度较高，企业经营困难加大，产业结构调整升级迫切，资源环境约束增加。

　　尽管今年的经济形势仍然复杂多变，但我们也要看到许多有利条件。全球共同努力挽救金融市场。我国发展总体上处于重要战略机遇期，经济发展向好的基本面没有改变，国家实施积极的财政政策和适度宽松的货币政策，推出扩内需、保增长、促就业一系列措施，力度之大前所未有。省委、省政府出台重要举措促进提高自主创新能力，促进传统产业转型升级，促进建设现代产业体系，保持经济平稳较快发展。改革开放30年我市建立了坚实的体制、物质、产业和社会基础，积累了应对复杂形势的综合实力，全市人民克服困难、力促发展的共识和力量坚定。

　　我们还要看到，国家实施《珠江三角洲地区改革发展规划纲要》，为我市实现新一轮改革发展带来新的重大机遇。我们要积极融入珠三角区域经济一体化，努力提高产业集群升级创新、统筹城乡发展综合改革的水平。

　　因此，我们要准确认识形势，正确对待困难。充分把握国际分工新一轮调整的有利时机；充分用好国家、省、市保增长促发展的政策措施；充分发挥全市上下尊重规律、坚韧不拔、团结奋斗的积极性和创造性。在市委的坚强领导下，我们完全有信心、有条件、有能力与全市人民一道，共克时艰，迎难而上。只要我们坚持科学发展，立足中山实际，振奋精神、科学谋划，求真务实、扎实工作，一定能够圆满完成今年各项目标任务，以优异成绩迎接新中国成立60周年。

　　2009年政府工作的总体要求是：高举中国特色社会主义伟大旗帜，坚持以邓小平理论和“三个代表”重要思想为指导，深入贯彻落实科学发展观，全面贯彻党的十七大、十七届三中全会和省委十届四次全会、市委十二届三次全会精神，以保持经济平稳较快发展为首要任务，着力于扩大内需保增长，着力于调整结构促转型，着力于改革创新增活力，着力于维护和谐惠民生，认真贯彻实施《珠江三角洲地区改革发展规划纲要》，加快转变发展方式，全面统筹城乡发展，在建设“三个适宜”新型城市道路上迈开新的步伐。

　　2009年经济社会发展的主要预期目标是：生产总值增长10%，单位生产总值能耗下降3.9%，二氧化硫和化学需氧量排放量分别下降5.4%和30.6%，全社会固定资产投资增长16%，社会消费品零售总额增长16%，外贸出口增长6%，地方一般预算收入增长10%，城镇居民年人均可支配收入增长6%，农民人均年纯收入增长6%，居民消费价格总指数升幅在4%左右，城镇登记失业率在3.5%以内，人口自然增长率在6.7‰以下。

2009年工作安排

　　2009年的工作重点是：保增长、扩内需、促转型、惠民生。就是在保增长上下功夫，把扩大内需作为保增长的根本途径，把促进发展方式转变、产业结构调整、经济结构转型作为保增长的主攻方向，把改善民生作为保增长的出发点和落脚点。

　　为实现上述目标，必须做好十方面工作。

　　一、加大投资消费拉动，确保经济平稳较快增长

　　促进投资稳定增长。充分发挥政府投资拉动作用，组织投入200亿元以上，安排88个重点项目，加大对交通能源、城建环保、社会事业、自主创新、重大产业、平安保障的投资力度。争取国家和省对我市重点项目支持，加强纳入国家重点和省新十大工程的项目建设。积极储备一批、招商引进一批、前期推进一批、开工建设一批、竣工投产一批重点项目。完善重点项目建设“绿色通道”配套政策体系，优化项目立项、招标等程序，落实重点项目责任制和进度督查。推行BT、BOT、代建制、收费质押、银团贷款、再担保、信托等融资方式，建立政府主导、市场参与、责权利相一致的多元投资体系。协调商业银行对重点项目给予贷款支持，激发社会投资活力。放宽住房公积金贷款政策，合理调整房地产相关规费，促进房地产市场稳定发展。

　　加强公共基础设施建设。开展“十二五”规划前期工作。启动新一轮以交通、电力、民生为重点的公共基础设施建设。实现嘉明电厂二期后续工程、500千伏桂山等输变电工程、北部组团垃圾综合处理基地一期、廉租解困房、东南门市场改造等项目年内投入使用。全面推进“五横六纵九加密”干线路网和“一港五区”建设，交通运输投入增长48%。加快建设新岐江公路、十水线、福源路、中山港二期、人民医院综合性公交枢纽站、社会福利院、黄圃和三角热电联产等项目。新开工建设东部快线、横二线、广珠中线二期、新图书馆、市档案中心和轻轨石岐站、小榄站、中山站及其配套路网等项目。推进小榄快线、番中大桥、纵一线、纵四线、横五线西段、广珠中线三期、古神公路二期、黄圃港、“139”文化街区等项目前期工作。

　　努力扩大消费。充分利用国家和省扩大内需政策，提高消费对经济的拉动作用。改善就业创业环境，努力提高居民收入，增强居民消费能力。培育新的消费热点，拉动农村市场，发展旅游、文化、休闲、健身等服务性消费和个性化消费。加快建设个人信用体系，扩大发展信贷消费。规范市场经济秩序，严厉打击生产销售假冒伪劣商品的违法行为，强化商品和服务质量监督，保障消费者合法权益。

　　保持价格水平稳定。强化价格监测预警和监督管理，完善公共产品和公共服务的价格形成机制。妥善处理价格调整对低收入群体和公益性行业的影响，保障低收入群体生活水平。认真落实价格补贴政策，安排公共交通政策性补贴2800多万元。完善价格信息披露机制，稳定市场预期。

　　二、推进产业结构优化，建设现代产业体系

　　积极发展先进制造业。坚持工业强市不动摇。加快工业高级化与适度重型化，努力发展先导性好、成长性高、支柱性强的重点产业，实现工业增加值增长10%。大力发展以装备制造、船舶制造、电子信息、光机电一体化为主导的制造业和高新技术产业，全面提升以家用电器、五金制造、灯饰光源、家具制造为优势的传统产业，积极发展以新材料、新能源、健康医药、游戏游艺为代表的新兴产业。力争2009年高新技术产品产值达1400亿元以上。提高产业组织整体实力，培育工业新的增长点。加快产业布局整合优化，推进产业集聚区建设。促进传统产业升级发展，实现从数量规模向效益优势转变。

　　大力发展服务业。优化升级传统服务业，发展金融服务、现代物流、商务会展、信息服务、文化旅游等现代服务业，实现服务业增加值增长12%。加大金融机构和金融产品发展步伐，鼓励各类金融、信用担保、风险投资机构发展。建设产权交易市场，发展法律会计、咨询策划、资产评估等中介行业。增强特色专业会展的国内外影响力，壮大会展经济。完善服务业外包产业链，发展粤港澳服务后台和外包基地。加快建设火炬区、东区、小榄等中心商务区，推进凤鸣、益华、富华、大信等特色商圈和轻轨站点经济圈建设。推进城区服务业错位发展，支持西区服务业综合改革试验区、石岐区火炬计划现代服务业特色产业基地建设。扶持小榄、三乡、坦洲、石岐等特色文化产业基地，发展文化产业。改造提升传统旅游项目，加快特色旅游发展，打造怡景片区等六大旅游板块，创建孙中山故里5A级国家旅游景区。加快服务业要素价格调整。

　　推进产业集群升级。建设“创新、开放、优质”的广东省产业集群升级创新试点，培养一批具有国内外影响力的区域品牌。推动产业集群延伸供应链和价值链，充分应用电子商务平台等渠道强化市场开拓能力，创新产业集群发展体系。促进装备制造、健康科技、信息服务等优势集群加快发展，建设好国家健康科技产业基地、华南现代中医药城、临海装备制造基地、软件园区；增强家用电器、家居安防、精细化工等传统集群竞争力，建设好小榄中国五金制品产业基地、南头中国家用电器制造基地、东凤中国小家电产业基地、黄圃中国家电产业配套创新基地；培育游戏游艺、卫浴洁具、花卉苗木等新产业集群，建设好港口中国游戏游艺产业基地、阜沙中国淋浴房产业基地、古镇南方绿博园。

　　增强发展后劲。鼓励企业抓住调整期机遇兼并重组，不断做大做强。推进大企业大项目带动产业发展模式，培育总部经济。以处于产业链高端、结构高级化、规模质量优势并举的大项目为重点，发展一批产值50亿元以上企业。推进中船工业船舶制造、中海油天然气利用、明阳风电设备、天乙铜材、嘉明电厂三期、南朗温泉城等项目建设发展。

　　帮扶企业渡难关。市财政扶持产业发展资金增至3亿元，引导企业重技术、树品牌、优结构、强管理、谋发展。市财政安排1亿元，实行中小企业贷款贴息、贷款风险补偿和担保补助。加强政银企合作，推进设立小额贷款公司试点。把握资本市场周期机遇，加大力度推进企业上市。支持风险投资及股权投资，拓宽企业融资平台。加强企业经营状况监测服务，建立多方预警和多种服务机制。

　　三、增强自主创新能力，提高经济发展质量

　　完善自主创新体系。市财政投入1亿元，实施“十大科技工程”。通过政府主导、资源整合、企业主体、市场运作，完善市、镇、企业三级技术服务网络。加强区域共性技术创新服务平台建设，为企业和区域产业发展提供支撑。推进科技成果孵化器、生产力促进中心、质量检验检测体系建设，筹建国家建筑金属制品质量监督检验中心。实施科技强企支撑计划，鼓励企业参与国家、省重大科技创新项目和省部产学研合作，自主组建或与科研机构共建工程技术研发中心。强化企业家创新意识，激励企业加大科技研发投入。

　　推进知识产权战略、技术标准战略和品牌带动战略。健全知识产权管理服务体系，加强知识产权保护和专利执法力度，完善重点行业专题专利信息数据库。积极与国际标准接轨，提升产品质量水平。推广“政府+行业协会+企业”联盟方式，发展技术规范和行业标准。重点培育中国名牌产品、驰名商标及出口名牌，促进产品经营向品牌经营转变。推动具有自主品牌、自主技术、自控市场的企业发展。

　　营造良好自主创新环境。加强科技创新政策配套，设立创新风险投资引导资金，建立多元化多层次自主创新投入体系。发展技术评估、成果交易、创新融资等服务机构。开展形式多样的科普活动，提高全社会科技意识和市民科学素养，鼓励创新创造。推进技能人才振兴计划，成立工业园区培训鉴定基地，建设有利于创新发展的产业人才体系。

　　四、扩大内外两个市场，提高经济开放水平

　　积极参与区域合作。把握广珠轻轨开通、深中通道规划建设等机遇，主动参与珠三角区域交通能源、公共安全、要素市场、产业协作、公共服务的一体化进程。加强与港澳在产业和金融领域的合作。建设好产业转移园区，有序推进产业和劳动力“双转移”。

　　加快转变贸易增长方式。优化对外贸易结构，推动进出口协调发展。市财政安排3800万元促进外贸增盈增效，鼓励企业进出口，重点扶持优势区域特色产品和有自主知识产权、自主品牌、附加值高的产品出口。发展一般贸易、服务贸易和技术贸易。开拓欧盟、东盟、拉美、中东等市场，构建多元稳定的国际贸易格局。扶助企业降低出口风险，鼓励企业使用出口信用保险工具，支持企业主动应对反倾销、反补贴等贸易壁垒。扩大资源能源、重要工业品和农产品进口。

　　促进加工贸易转型升级。推动加工贸易企业原地不停产转型扩大内销，发展一批面对国内需求的专业市场。鼓励加工贸易向上下游延伸，引导企业增资扩产、扩大国内采购和深加工结转业务。完善准入管理，引进技术含量和增值率高的加工制造环节。建设用好保税物流中心、保税仓等海关特殊监管区域。鼓励企业参与各类展销会，发展国内外销售网络。推进加工贸易联网监管，完善“大通关”建设。

　　提高招商引资质量。加强对国际资本和市场的研究跟进，开拓新的外资来源地。优化利用外资结构，加大对国际龙头企业、央属企业、国内上市企业、省属大型国有企业和优秀民营企业的招商力度。引进先进技术和管理经验，鼓励跨国公司在本市设立地区总部、研发中心和采购中心，促进外商投资向研发设计、核心部件生产和物流配送等环节延伸。推进能源环保项目的国际合作发展。发挥商会、行业协会在招商引资中的积极作用。

　　五、加快农村改革发展，推进城乡一体化建设

　　提升城乡规划建设水平。加快建设社会主义新农村，促进农民增收、农业增效、农村发展。推进城乡规划、基础设施、公共服务、社会保障、社会管理的一体化建设。新增强农惠农投入6900万元，提高财政保障农村各项事业水平。完成村级规划编制，合理安排城镇建设、农田保护、产业聚集、村落分布、生态涵养、文物保护等空间布局。促进城乡产业加快融合，区域配套均衡发展。稳步推进“村改居”和城中村改造，建设农民居住小区，优化农民生活环境。以加大投资拉动为机遇，加强农村基础设施建设。提高农村公路建设等级标准，健全农村公路管护机制。建设220个农村候车亭，加快农村出行公交化步伐。完成250公里主干农路硬底化，建设2.6万亩连片耕地的农路农桥，建设标准农田和改造中低产田1.2万亩。全面落实城乡水利防灾减灾任务，完成中顺大围达标加固和西河水闸工程，启动中珠联围水闸重建，提高防洪抗风险能力。

　　逐步实行城乡统一的公共服务。安排1.2亿元实行财政转移支付。加强农村卫生基础设施建设，改善农村预防保健和医疗救治条件。建立政府扶持、多方参与、市场运作的农村信贷担保机制，扩大政策性农业保险覆盖面，完善农业风险救助机制。鼓励农村建立社区综合服务中心。建立与经济社会发展相适应、城乡统筹的社会保障体系。做好被征地农民社会保障工作。

　　加强农村社会管理。规范农村土地管理制度，促进农村土地产权明晰、节约集约和科学管理。推行农村土地承包经营权流转，落实农民对承包土地的使用权和收益权。推进农村集体土地管理改革，建立城乡统一的建设用地市场。城管执法工作向村（居）延伸。加快村级统一核算改革。发展新型村级集体经济组织，推动向股份有限公司过渡。壮大农村集体经济，实现所有欠发达村集体经济年纯收入100万元以上。规范农村集体经济组织和资产财务管理，加强村级审计监督，完善农村基层干部激励保障机制。

　　积极发展现代农业。提高农业市场化、专业化、规模化水平，鼓励农产品精深加工，发展农产品专业市场，支持港口、民众等镇发展农业物流和生态农业。提高农民生产经营组织化程度，培育壮大农业龙头企业。加大农业科技投入，深化镇区农业技术推广体系改革，推进农业机械化。扶持花卉苗木、特种水产养殖等向优势产业发展，大力建设农业现代园区。培育绿色食品、无公害农产品、农业名牌等农产品标识，推行标准化生产和农产品质量认证，打造一批生产示范基地。加强动植物疫病防控，推动疫病禽畜无害化处理。确保粮食生产稳定。加强海洋资源环境保护及海域管理。

　　六、坚持节约能源资源，加强生态环境建设

　　全力创建全国生态市。加大环保基础设施建设力度，市环保投入增长15%。启动建设第二批10万吨污水处理厂，加快城区珍家山污水处理收集系统二期、镇区生活污水处理厂及其配套管网建设，提高城镇生活污水处理率。推进电厂脱硫工程，加快固体废物综合处理中心、南部组团垃圾综合处理基地建设，生活垃圾无害化处理率为100%。总投入3600万元建设大气环境、江河水质和机动车尾气在线监控系统，完善重点污染源监管，完成污染源普查。全部镇创建成为全国环境优美乡镇，开展农村小康环境生态示范活动。加强水资源管理，投入1.5亿元进行内河整治。加强五桂山自然生态保护，推进“绿化大地工程”，完成林相改造5400亩。力争通过全国生态市验收。

　　加强节能降耗工作。全面落实污染减排工作责任制，建立市镇两级污染减排协调机制。完善节能减排评估体系和考核制度，加强重点能耗及污染企业的监管和指导。加大违法排污企业查处力度，严厉查处环境违法行为。深入推行清洁生产，加快淘汰落后生产能力，改善产业能源供应结构。弘扬生态文化，构建政府大力推进、市场有序调节、企业自觉行动、公众积极参与的节约型社会。

　　推进节约集约用地。全面开展土地预征预控。严格土地管理，加快新一轮土地利用总体规划修编，做好园地、低坡山地等补充耕地指标工作。加快“三旧”资源改造利用步伐，加大闲置用地处理力度。健全土地监管长效机制，落实耕地保护共同责任机制，严厉查处整治违法违规用地行为。加强矿产资源利用管理，做好防治地质灾害工作。

　　七、科学依法治税理财，推动公共服务均等化

　　保持财政收入稳定增长。坚持依法治税，大力培育税源和新的税收增长点。完善收入异动预警机制，确保应收尽收。认真落实增值税转型等税收政策，不断拓宽收入领域。统筹政府非税收入，扩大“收支两条线”管理范围，强化土地及规划收费征管，促进政府收入增加。加强政府经营性资产资源管理。

　　优化财政支出结构。按照保运转、保重点、保民生和集中财力办大事的原则，优化财政资源配置。安排59%财力用于公共服务和公共产品，新增财力重点投向扶持企业发展、基础设施建设和公共社会事业，其中69%用于提高教育、医疗、社保等公共服务产品保障水平。坚持过“紧日子”，严格压缩和控制一般性支出，实施行政经费零增长，专项经费减5%。合理安排预算支出，严格控制追加预算。创新政府项目投融资体制，减轻财政支出压力，确保财政安全。

　　深化财政管理体制改革。完善激励型财政分配体制，健全市镇财政收入划分、转移支付的稳定机制。完善政府采购制度，扩大国库集中支付范围。加强财政支出绩效评价和专项资金管理，强化预算执行审计和重点专项资金审计。

　　完善国有资产管理体制。深化市属企业用人、薪金制度改革，提高国有资产依法监管水平。加快国有企业业务整合，向优势领域扩展。加强国有资产经营预算、业绩考核、收益收缴、资产转让和产权管理。清理盘活闲置国有资产，增强市属集团公司融资发展能力。放宽市场准入，引进社会资本和竞争。

　　八、繁荣社会各项事业，加快建设文化名城

　　坚持优先发展教育事业。市财政投入23.7亿元，加快教育现代化建设步伐。促进基础教育均衡优质发展，推进振兴初中行动计划、学校优质化工程和名校带动战略。高质量高水平普及高中教育，新增高中教育市级成本分担2300万元，落实“生均经费市镇共担”机制，实施“扩容促优”工程，扩大品牌高中办学规模。大力发展职业技术教育，支持职业院校与企业合作办学，推进学校后勤社会化。支持电子科技大学中山学院、广东药学院中山校区、广东理工学院等提升高等教育水平。促进优质民办教育和成人教育健康发展。完成中山职业技术学院三期、市实验高中扩建、中山中专二期、第一职业技术学校、高级技工学校黄圃校区一期等项目，推进华侨中学高中部、职业技术教育实训基地等建设。坚持人才强市战略，完善培养、吸引、留住、用好人才的政策措施，突出加强技能人才队伍建设，为经济社会发展提供人才智力支撑。

　　全面推进文化名城建设。实施孙中山文化等“八大文化工程”，完善公共文化服务体系。加快重点文化设施建设，全面完成农家书屋建设任务。创建国家历史文化名城，加强文化遗产保护利用，扶持镇区创建国家级、省级民族民间艺术之乡。扶持培育影视动漫、工业设计、演艺策划等创意产业。提升特色产业的文化内涵和价值。深入开展精神文明创建活动，促进人的全面发展。

　　加快医疗卫生事业发展。加强公共卫生服务体系建设，完善市、镇、村三级公共卫生服务网络。按照预防、保健、康复、健康教育、计生服务和基本医疗等“六大功能”要求，推进城乡社区卫生服务规范化建设。加强镇村医疗卫生一体化管理，提高基层综合卫生服务能力。探索医疗卫生体制改革，构建和谐医患关系。力争2010年实现全民医保，达到“小病在社区、大病到医院、康复回社区”的目标。完成新中医院工程，加快中心血站等项目建设。巩固爱国卫生运动成果，加强传染病防控。让市民享有安全、有效、方便、价廉的医疗卫生服务。

　　积极推进体育、人口计生等工作。广泛开展群众性体育运动，举办市第六届运动会，加强镇村体育设施建设。健全人口计生层级动态管理和利益导向机制，稳定低生育水平，提高出生人口素质。探索建立外来人口积分服务管理机制，科学控制人口规模。扎实推进对口援建汶川县漩口镇工作。做好外事、侨务、港澳事务、台务、民族、宗教、妇女儿童、老干、统计、人防、气象、地震、档案、地方志等工作。

　　九、大力改善民生保障，促进社会和谐稳定

　　推进社会管理体制改革。降低准入门槛，实施财政扶持，大力发展社会组织，构建社会管理主体多元化格局。完善社会管理网络，提高各类组织的自律性、诚信度和规范化水平。推行公共服务市场化改革，健全政府购买公共服务机制。探索新的社区治理与服务模式，推进农村自治组织建设，开展农村社区建设试点工作。

　　促进城乡居民就业创业。市财政投入2500万元推进就业创业。加大社区（大嫂）工作坊、企业村（社区）车间等就业基地建设力度，鼓励农村劳动力就地就近就业。完善公共就业政策及服务体系，新增就业岗位4万个。深入开展创业带动就业工作，探索建立创业孵化基地。启动失业基金3000万元帮扶失业人员培训和再就业。加强在职人员岗位培训、下岗人员和农村富余劳动力就业创业培训、免费职业技能培训，培训城乡劳动者5万人次以上，确保每个农村家庭都有具备就业能力的劳动力接受职业技能培训。

　　完善社会保障机制。市财政投入2.6亿元用于社会保障。提高城镇职工社会保险覆盖面，参保总人次达470万。实现农村基本养老保险全覆盖，逐步提高企业退休人员养老待遇，关心困难群体社会保障。加快推进城乡居民医疗保险一体化，城乡居民门诊基本医疗保险覆盖率达80%以上。安排3100万元用于城乡社会救济和最低生活保障，完善以最低生活保障为基础，以医疗、住房、教育、就业、司法、临时救助等为辅助的社会救助体系。围绕监测预警、物资储备、社会动员、舆论引导等重点环节，加强灾害应急救助工作，增强防灾抗灾能力。健全住房保障制度，市财政投入1.2亿元加快廉租房供应，推行以实物配租和租金补贴相结合的廉租房保障体系。完成低保家庭危房改造和城区预制构件公房改造。完善双拥优抚安置工作体系，探索退役士兵安置一体化政策。发展社区公益类社会组织和慈善公益组织，推动老龄事业发展，做好残疾人工作，提高孤残儿童养护水平，发展慈善福利事业。

　　维护社会和谐稳定。建设适应社会转型的利益协调、诉求表达和权益保障机制，构建以人民调解为主线的大调解格局，完善信访工作网络。规范各类群体性事件处置方式，健全突发公共事件应急机制。依法维护劳资各方合法权益，构建和谐劳动关系。加强社会治安综合治理，实施公安视频监控系统二期工程，健全社会治安防控体系。完善打击各类违法犯罪的长效机制。加强互联网管理，推进“网防工程”建设。关心流动务工人员，加强服务管理机制建设，提高流动人口服务管理效能。加强安全生产、交通安全、消防安全、食品安全、医药安全等工作，构建平安和谐社会。

　　十、加强政府自身建设，提升行政服务水平

　　推进行政管理体制改革。按照中央和省的部署，以建立职能有机统一的大部门体制为重点，开展行政机构改革工作。完善行政部门内部决策权、执行权、监督权既相互制约又相互协调的运行机制，努力做到权责一致、分工合理、决策科学、执行畅顺、监督有力。推进事业单位分类管理，规范机关事业单位聘用人员管理制度。

　　继续转变政府职能。全面推进政企分开、政资分开、政事分开、政府与市场中介组织分开，把不该由政府管理的事项转移出去，把该由政府管理的事项切实办好。注重社会管理和公共服务，着力解决好人民群众关注的重点难点热点问题。扩大政府公共服务覆盖范围，提高公共服务供给能力和质量。

　　坚持依法行政。自觉接受人大的法律监督和工作监督，认真落实人民代表大会及其常委会的决议。主动接受人民政协的民主监督，广泛听取民主党派、工商联、无党派人士和各人民团体的意见。提高议案、建议和提案办理质量。巩固扩大基层民主，推动政府依法管理与基层群众自治良性互动。贯彻行政许可法，落实依法行政实施纲要，严格按照法定权限和程序履行职责。加强政府法制建设，规范行政行为，坚持用制度管权、管事、管人。正确行使行政处罚自由裁量权，确保行政执法公开、公平、公正。建立行政复议委员会制度。深入开展普法教育，做好法律服务和法律援助工作。

　　优化政务环境。深化行政审批制度改革，推进行政效率提速。扎实开展广东省“创新电子政务服务平台，提高政府办事效率改革试点”工作，推行重点项目协同办公、三级电子监察、行政服务热线、网上投诉调处等电子政务。畅通政府与群众双向沟通渠道，坚持问政于民、问需于民、问计于民，切实维护群众利益。加快推进政务信息公开，提高政府工作透明度和公信力，充分保障人民群众的知情权、参与权、表达权、监督权。

　　建设廉洁政府。深入开展学习实践科学发展观活动，在政府机关形成勤奋学习、真抓实干、艰苦创业、为政清廉的风尚。坚持求真务实，改进文风会风。加大监察力度，从源头上预防和治理腐败。深化经济责任审计，推进廉政文化建设。树立行政成本意识，厉行节约，勤俭办事，做清正廉洁的表率。加强对政府公务员的教育、管理和监督，建设高素质的公务员队伍，建设广大人民群众满意的政府。
